# Gastrointestinal Platyhelminths of Free-Living *Cuniculus paca* (Linnaeus, 1766) in the Western Amazon

**DOI:** 10.1590/S1984-29612024069

**Published:** 2024-11-22

**Authors:** Victor Silva Vasconcelos, Maria Isabel Afonso da Silva, Rodrigo Lima do Nascimento, Matheus Nascimento Oliveira, Rodrigo Cacique Rocha, Manuela Jucá Correia, Cledson Kauã Araújo Silva, Wendell Nogueira Dias, Felipe Bisaggio Pereira, Iago de Sá Moraes, Dirceu Guilherme de Souza Ramos, Tiago Lucena da Silva

**Affiliations:** 1 Programa de Pós-graduação em Sanidade e Produção Animal Sustentável na Amazônia Ocidental – PPGESPA, Centro de Ciências Biológicas e da Natureza, Universidade Federal do Acre – UFAC, Rio Branco, AC, Brasil; 2 Departamento de Parasitologia, Instituto de Ciências Biológicas, Universidade Federal de Minas Gerais – UFMG, Pampulha, Belo Horizonte, MG, Brasil; 3 Programa de Pós-graduação em Biociência Animal – PPGBA, Instituto de Ciências Agrárias, Universidade Federal de Jataí – UFJ, Jataí, GO, Brasil

**Keywords:** Game animals, Amazonia, Cestoda, Trematoda, Animais cinegéticos, Amazonia, Cestoda, Trematoda

## Abstract

Studies on Brazil’s helminthological fauna began in the early 20th century, and since then several zoologists from different parts of the country have focused on parasitology. Recent studies have advanced our understanding of helminthological biodiversity in the Amazon region, especially in fish, which is essential for the Amazonian economy. This study aimed to inventory the platyhelminth fauna *Cuniculus paca* (paca). The viscera of 30 pacas were analyzed, and 60 specimens of platyhelminths were identified, including 52 cestodes and 8 trematodes. Cestodes belonging to the family Davaneidae, identified as *Raillietina* spp., with an occurrence of 26.67% (n = 8), mean abundance 1.83 and mean intensity of 6.5, were found in the small intestine. The trematodes found in the large intestine were identified as *Stichorchis* spp., with an occurrence of 6.6% (n = 2), mean abudance 0.32 and a mean intensity of four. To the genus *Raillietina*, we provide new insights into the helminth fauna of this game rodent in the Western Amazon. The discovery of a new site of occurrence for the genus *Stichorchis* highlights the gap in knowledge regarding the parasitic diversity of consumed animals in the extreme western Amazon region, emphasizing the need for more in-depth scientific investigations in this area.

## Resumo

Os estudos sobre a fauna helmintológica do Brasil começaram no início do século 20 e, desde então, vários zoólogos de diversas partes do país têm se concentrado na parasitologia. Estudos recentes avançaram na compreensão da biodiversidade helmintológica na região amazônica, especialmente em peixes, essenciais para a economia amazônica. Este estudo teve como objetivo inventariar a fauna de platelmintos em *Cuniculus paca* (paca). Foram analisadas as vísceras de 30 pacas e identificados 60 espécimes de platelmintos, sendo 52 cestódeos e 8 trematódeos. Os cestódeos, pertencentes à família Davaneidae, foram identificados como *Raillietina* spp., com ocorrência de 26,67% (n = 8), abundância média de 1,83 e intensidade média de 6,5, encontrados no intestino delgado. Os trematódeos, encontrados no intestino grosso, foram identificados como *Stichorchis* spp., com ocorrência de 6,6% (n = 2), abundância média de 0,32 e intensidade média de quatro. Para o gênero *Raillietina*, foram fornecidas novas informações sobre a helmintofauna desse roedor cinegético na Amazônia Ocidental. A descoberta de um novo local de ocorrência do gênero *Stichorchis* destaca a lacuna no conhecimento sobre a diversidade parasitária de animais, consumidos no extremo oeste da Amazônia, enfatizando a necessidade de investigações científicas mais aprofundadas nessa área.

## Introduction

Studies on the helminthological fauna of Brazil began with research by Pirajá da Silva and Gomes de Faria in the first decade of the 20th century, initially focusing on *Schistosoma mansoni* (Travassos et al., 1969). Subsequently, Travassos continued a series of investigations in the early second decade of the century (Travassos et al., 1969). In the first half of the 20th century, various researchers significantly advanced parasitology, expanding our knowledge of Brazilian helminthofauna. Among these researchers were Viana, Lutz, Vaz, Pereira, Artigas, Teixeira de Freitas, Lent, and Machado Filho (Noronha et al., 2009).

Vicente et al. (1997) pioneered the identification of helminths in wild mammals, providing one of the most comprehensive identification keys for this group. Helminthological studies in the Amazon have substantially progressed. Research by Oliveira et al. (2020) and Silva Lima et al. (2022) has greatly contributed to the knowledge of helminth biodiversity in fish in the region, an extremely relevant factor given the cultural context in which fish are not only a primary source of protein but also play a crucial economic role (Corrêa et al., 2016).

Additionally, the consumption of wild animals in the Western Amazon is significant, especially among traditional, riverside, and settled populations that frequently rely on game as their main food resource (Chaves et al., 2018; Lemos et al., 2018). Among the game animals in the region, certain rodents, particularly the paca (*Cuniculus paca*), are a popular element in the local cuisine and frequently consumed. The paca has also been targeted for commercial breeding in this region (Ribeiro et al., 2016).

Studies on the helminth fauna of *C. paca* remain scarce in the Amazon. Some research reports the occurrence of polycystic hydatid disease in this rodent, highlighting the zoonotic risk posed by *Echinococcus vogeli*, which is also responsible for causing the condition known as 'paca disease' in humans. Meneghelli et al. (1990) and Pastore et al. (2003) reported *C. paca* as the intermediate host and humans as accidental hosts.

Considering the high consumption of wildlife in this region and the significant gap in knowledge regarding their parasitology, particularly in light of reports of zoonotic parasites affecting both wildlife and local populations, this study aimed to investigate the platyhelminth fauna of *C. paca.*

## Materials and Methods

The study material was obtained from the Paranã da Floresta community, located in the municipality of Guajará, Amazonas. This area, which is inhabited by traditional communities, is not included in any classification in the National System of Conservation Units (SNUC). The residents of Paranã da Floresta have distinctive ways of life, similar to those of other traditional Amazonian peoples, being mainly composed of extractivists and riverine populations. The local economy is based on the production of manioc flour, the commercialization of fishery resources, often extracted from the Paranã River, which gives the community its name, and family farming.

The research project was supported by the residents of the Paranã da Floresta community. Whenever possible, after the animals were killed for consumption, the viscera were removed, packaged separately per animal in plastic bags, and stored in a freezer. The residents informed the research team, and so, the research team traveled to the site to collect the discarded viscera.

Platyhelminths were recovered through analysis of each organ to identify the sites infected by parasites. The organs were dissected, and the contents were processed using a Tamis sieve (0.15 mm) and evaluated in Petri dishes using a Zeiss Stemi 508 stereomicroscope (10x magnification). Parasites were processed as described by Amato (1985) and identified as described by Khalil et al. (1994), Gibson et al. (2002), and Jones et al. (2005). The occurrence, mean intensity, and mean abundance data were calculated according to the methods described by Bush et al. (1997).

## Results

The viscera of 30 pacas were recovered, revealing a total of 60 platyhelminths: 52 cestodes in eight pacas and eight trematodes in two pacas (Table 1).

**Table 1:** Platyhelminth species found in *Cuniculus paca* in Western Amazon with data on occurrence, average abundance, average intensity and total number of individuals.

**Species**	**Site of infection**	**Ocurrence**	**Mean Abundance**	**Mean Intensity**	**Total helminths**
**Cestodes**					
*Raillietina* spp.	Small Intestine	26.67	1.83	6.5	52
**Trematodes**					
*Stichorchis* spp.	Large Intestine	6.6%	0.32	4	8

The cestodes in the small intestine were found to be from the Davaneidae family, with an occurrence rate of 26.67%, a mean abundance of 1.83, and mean intensity of 6.5 worms per infected paca.

The cestode count was established only after the scolices had been identified. Numerous strobilae and proglottids were found; however, the count was performed only after locating the anterior region of the parasite.

**Figure 1:**
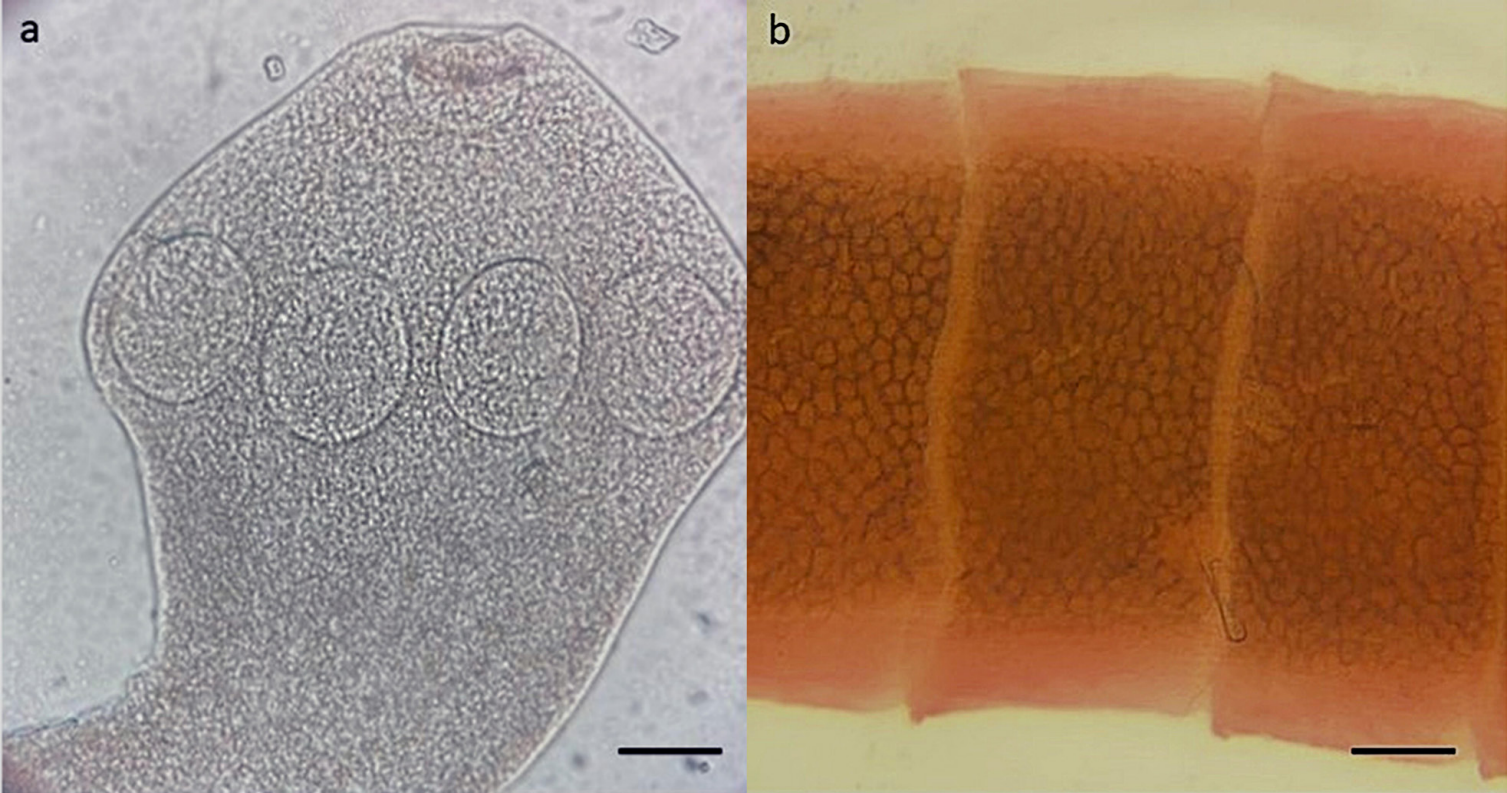
Morphological characteristics of *Raillietina* spp. found in *Cuniculus paca* from Western Amazon. (a) Small scolex with four suckers and rostellum armed with hooks. Scale bar = 0.25 mm (b) Pregnant proglottids containing multiple egg capsules. Scale bar = 0.8 mm.

The most important characteristics for classification at the genus level were a small scolex with four suckers and a rostellum armed with hooks (Figure 1a). Proglottids craspedote, with immature and mature proglottids wider than long. The female reproductive system consists of a bilobed ovary and a posterior vitellarium. Gravid proglottids contain multiple egg capsules (Figure 1b), and small spines may be present on the suckers, which suggests the diagnosis of *Raillietina* spp.

Eight trematodes located in the large intestine were identified as *Stichorchis* spp. Trematodes occurred in 6.6% of the analyzed hosts, with a mean abundance of 0.32 and a mean intensity of four individuals per host.

**Figure 2:**
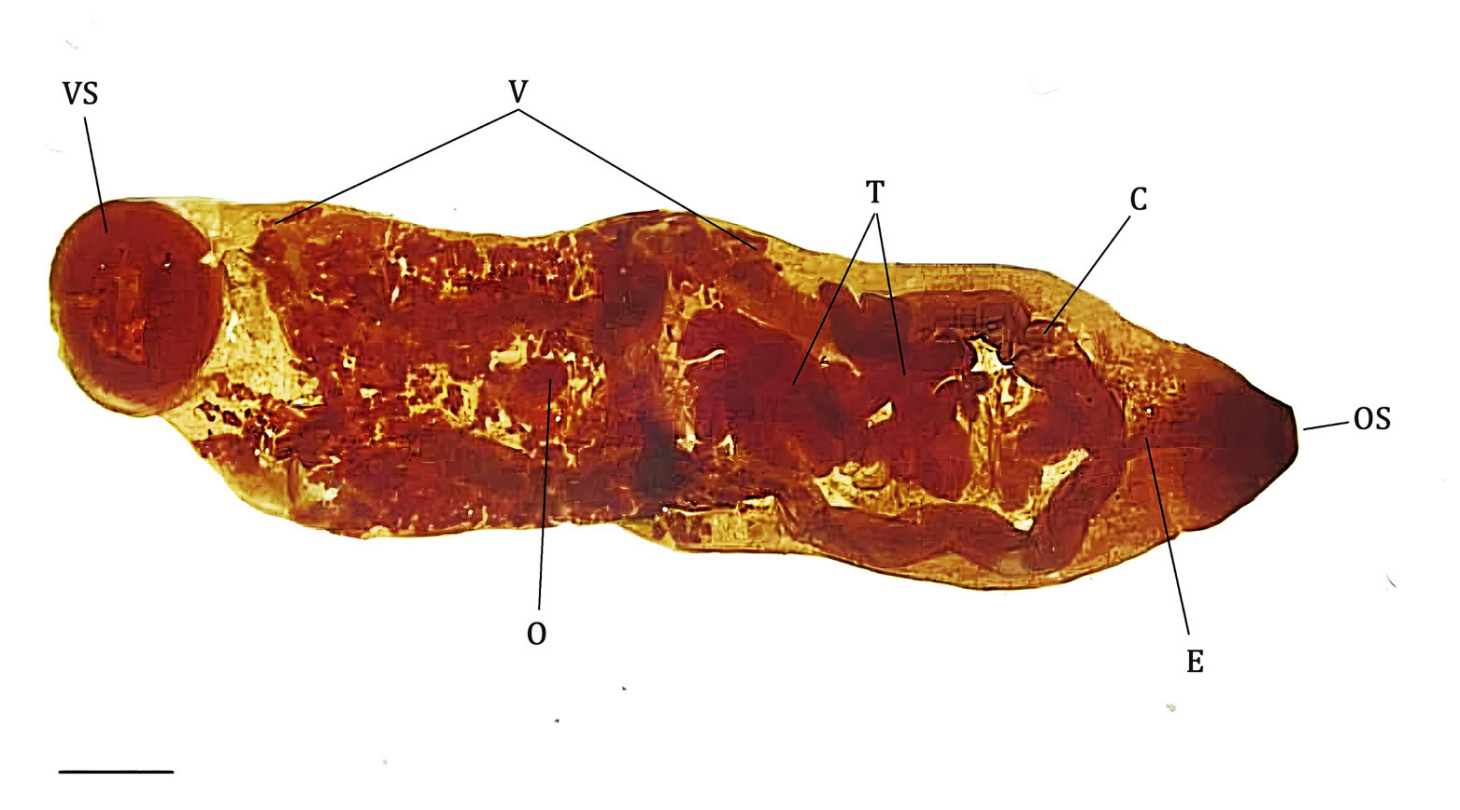
Morphological characteristics of *Stichorchis* spp. found in *Cuniculus paca* from Western Amazon. OS=Oral sucker; E=Esophagus without a bulb; C=Cecum; T=Testes in tandem between the ceca; V=Vitelline follicles anterior to the acetabulum; VS=Ventral sucker/Acetabulum is ventro-subterminal; O=Ovary; Genital sucker was observed only under a stereoscopic microscope into the eophagus/intestine junction. Scale bar = 1mm.

Identification of the genus *Stichorchis* was based on the morphological criteria described in the literature. Trematodes have larger bodies than other groups of platyhelminths found in *C. paca*, with an oval-elongated shape. The acetabulum is a ventro-subterminal region, and the esophagus lacks a bulb. The testes are arranged in tandem, that is, one after the other in the middle third of the body, and are deeply branched, with the ends of the lateral branches partially overlapping the intestinal ceca. The genital sucker has a genital sphincter. The ovary is located anterior to the acetabulum, with a dorsal Mehlis gland. The vitelline follicles are located in the lateral fields from the bifurcation level to near the acetabulum without overlapping. The uterus forms transverse spirals in a medially limited field containing numerous eggs (Figure 2).

The genus *Stichorchis* consists of two known species, *S. giganteus* and *S. subtriquetus*, with only *S. subtriquetus* having morphometric data described in the literature. Table 2 presents the morphometric variations observed for *S. subtriquetus* and the *Stichorchis* species reported in the present study.

**Table 2:** Morphometric data of *Stichorchis* spp. of *Cuniculus paca* from Western Amazon, compared to *Stichorchis subtiquetus* collected in the Czech Republic. Measurements in μm.

**Parasites**	***Stichorchis* spp.**	***Stichorchis subtriquetus* (** **Benovics et al., 2022** **)**
Country	Brazil	Czech Republic
Number of specimens	8	45
Total length	17,498.07	9,868
Maximum width	4,836.53	4,516
Ventral sucker length	2,191.37	2,315
Width of the ventral sucker	2,535	2,249
Pharynx length	1,112.29	1,165
Length of the esophagus	841.68	468
Testicle length	3,218.53	2,291
Width of the anterior testicle	2,247.75	2,518
Ovary length	771.28	560
Ovary width	556.25	633

## Discussion

The low number of cestodes found was due to the method of sample storage and screening. After the freezing process, it is common for cestodes to fragment, which complicates the visualization of scolices. Cestodes of the family Davaineidae have been reported in rodents from various regions of the world (Guerreiro et al., 2023). Although these are common parasites, it is difficult to collect intact specimens in large numbers, and quality of material is critical for identification. Beveridge & Smales (2017) reported the occurrence of Davaineidae in New Guinea and adjacent islands but faced difficulties in finding intact and well-preserved organisms.

Simões et al. (2017), in a morphological and molecular analysis of the genera *Fuhrmannetta* and *Raillietina* from the family Davaineidae, argue that their morphologies are similar and combine them into *Raillietina*. *Raillietina* is characterized as a parasite that causes significant problems in animal production. The hosts present with mucosal lesions, damage to secretory glands, epithelial cell sloughing, reduced glycogen levels, and weight loss, making the parasite a significant threat to animal health. Such damage negatively affects the potential economic exploitation of *C. paca* in the extreme western Amazon region, as proposed by Ribeiro et al. (2016).

Costa et al. (2022) described the genus *Raillietina* as a parasitic infectant in sigmodontine rodents belonging to the Oryzomyini tribe, which includes approximately 30 genera. The authors suggested that taxonomic similarity between genera may be a predominant factor in the occurrence of this parasite. This observation reinforces the hypothesis that *Raillietina* significantly infects rodents.

Trematodes have a leaf-like body shape with two suckers, a ventral sucker and an oral sucker, for attachment to host tissues, and a well-developed reproductive system, both of which are easily visible. The initial identification at the family level was made following the observation of key morphological structures. The body is large, conical, and dorsoventrally flattened, with an acetabulum located at the posterior end. Pharyngeal sacs are muscular and prominent, and a long esophagus with a terminal bulb is present. Two entire and branched testes are located between the ceca, slightly overlapping and arranged in tandem. An external seminal vesicle is typically present, as well as a genital sucker. The ovary is positioned post-testicularly. Laurer's canal opens dorsally. The intercecal uterus possesses a small portion that laterally crosses one of the ceca, and numerous eggs are observed. The distribution of vitelline follicles is extensive, spanning the length of the body up to the acetabulum (Jones et al., 2005).

The literature reveals a significant gap in morphometric data for species of the genus *Stichorchis*, which currently comprises only two described species. Comparing with the morphometric data for *Stichorchis subtriquetus* reported by Benovics et al. (2022), some discrepancies in the dimensions of certain structures have been observed. Regarding the second species, *S. giganteus*, no morphometric data are available in the literature. However, further studies are needed to confirm whether these specimens are a new species or a variation of existing species.

Stunkard (1925) proposed that the observed morphological characteristics would justify elevating this group to the full genus status, removing its previous classification as a subgenus. Belonging to the family Cladorchiidae, the genus *Stichorchis* currently includes two species, *S. giganteus* and *S. subtriquetrus* (Jones et al., 2005). The species described in this study presents some characteristics that differ from the two known species but retains all characteristics of the genus, suggesting the possible existence of a new species within the genus *Stichorchis* and highlighting the relevance of studies of this nature, given the limited information on the genus *Stichorchis* in the literature.

The rodent *Dasyprocta punctata* is phylogenetically close to the host in this study, and *S. giganteus* has been found in *D. punctata* in Colombia (Flores-Peredo et al., 2020), as well as in Trinidad and Tobago (Jones & Garcia, 2021). The identification key used for the parasite specimens was from Jones et al. (2005), who described the occurrence of this genus in the Dasyproctidae. However, the characteristics of individual size, positioning, conformation of the testes, and the positions of the vitelline follicles differed from those given in the identification key.

The nearest known record of *S. giganteus* to the study area was in Bolivia, where the parasite was found in *Pecari tajacu*, an animal also present in the study area (Limachi Quiñajo et al., 2014). However, the genus *Stichorchis* had not yet been described in the extreme western part of the Amazon in Brazil. Indeed, this report is not only the first for this genus in the region, but may also be the first record of a new species within the *Stichorchis* genus.

As mentioned, Meneghelli et al. (1990) and Pastore et al. (2003) describe the occurrence of *E. vogeli*, a parasite with zoonotic risk occurring in pacas. Unfortunately, this is an organ highly valued for consumption by the community. This fact resulted in the non-donation of viscera for analysis without the inclusion of the liver, making its observation impossible.

## Conclusion

The platyhelminths identified in *C. paca* exhibited a lower occurrence, abundance, and mean intensity than nematodes previously described in the literature for the same host. This underscores the need for further studies in the Amazon, as data on platyhelminths in wild animals from this region are scarce. The genus *Raillietina*, although well documented in scientific literature, provides new insights into the helminth fauna of this game rodent in the Western Amazon. Although there have been studies on helminths in animals consumed, none have been conducted in the region in question to date.

The discovery of a new occurrence site for the genus *Stichorchis* highlights the gap in knowledge regarding the parasitic diversity of animals consumed in the extreme western Amazon, underscoring the need for more in-depth scientific investigations in this region. The development of detailed morphological and molecular studies will aid in understanding the biology of these parasites, enabling the correlation between the damage inflicted by the parasites and its impact on the weight or function of the host organism.
